# Uncertainty analysis of a non-automatic weighing instrument from calibration data on scales according to the SIM guide

**DOI:** 10.1016/j.dib.2020.106436

**Published:** 2020-10-20

**Authors:** Germán Roca-Gómez, Ulises Ospino-López, Cristian Antonio Pedraza-Yepes, Oscar Fabián Higuera-Cobos, José Daniel Hernández-Vásquez

**Affiliations:** 1 Universidad del Atlántico, Faculty of Engineering, Mechanical Engineering Program, Research Group CONFORMAT, Puerto Colombia, Colombia; 2 Universidad Antonio Nariño, Faculty of Mechanical, Electronic and Biomedical Engineering (FIMEB), Research Group GIFOURIER, Mechanical Engineering Program, Puerto Colombia, Colombia

**Keywords:** Calibration, Measurement uncertainty, Metrology, SIM guide

## Abstract

This research was motivated by technical-economic challenges imposed by mass metrology, specifically, in matters concerning calibration methods of non-automatic type weighing instruments (*i.e.*: digital scales). In order to contextualize the problem detected, in the industry there are different processes of mass measurement that are controlled by digital scales, such as: mass of liquids, chemicals, food, body mass of a person. In these processes, the scale is used in the following four conditions for mass measurement: (i) ascending and descending load, returned to zero; (ii) ascending and descending load, without the need to return to zero; (iii) only with ascending load and (iv) only with descending load. In this context and, maintaining the principles for the calibration of a measurement instrument in which it must be carried out under the same operating conditions as the instrument, metrology laboratories must knowing the metrological reliability (*i.e.*: errors and uncertainties) for each situation. This is exactly the main motivation for the development of the research. Thus, the experimental data obtained in a research laboratory under controlled environmental conditions allowed obtaining a minimum expanded uncertainty associated with the mass measurement of 0.0012 kg (*k*=2; confidence level: 95%).

## Specifications Table

**Table:** 

Subject	Mechanical engineering.
Specific subject area	Measurement of physical magnitudes (temperature, pressure, and mass) applied in Mechanical Engineering.
Type of data	Table.
How data were acquired	Experimental measurements performed in a Nutrition and Dietetics Laboratory of the Universidad del Atlántico under specified environmental conditions. To develop the experiments and guarantee the metrological reliability of the data, a non-automatic weighing instrument (*i.e.*: digital scale) was used (range: 0 to 30 kg; resolution: 0.001 kg). A calibrated digital barometer with a resolution equal to 0.1 mbar/abs. For the measurement of ambient temperature, a calibrated digital thermometer with a resolution of 0.1 °C was used. For the calibration of the scale, a class E1 standard mass set was used according to the OIML R-111-1:2004 classification.
Data format	Raw and analysed.
Parameters for data collection	The data were acquired in the Nutrition and Dietetics Laboratory of the Universidad del Atlántico (Barranquilla-Colombia) under controlled environmental conditions. The ambient temperature ranged between 27.4 °C and 27.7 °C. The atmospheric pressure was kept constant at 1021.7 mbar/abs. This ensured uniform air density and, additionally, a constant buoyancy force throughout the process. To start the measurement process, a period of 30 minutes was waited for the thermal equilibrium between the standard masses and the atmospheric air to be established. In this way, undesirable variations in the mass indication due to the effects of convection heat transfer were avoided. Special care of vibration and atmospheric air currents were considered in order to avoid them. Finally, once all the parameters that influence the mass measurement process were controlled, it was possible to start with the collection of experimental data.
Description of data collection	The scale calibration was carried out by applying four different methods: (i) ascending and descending load, returned to zero; (ii) ascending and descending load, without the need to return to zero; (iii) only with ascending load and (iv) only with descending load. This was done with the objective of evaluating the potentiality and applicability of each one as appropriate in each case. A total of 42 experimental data were collected during the mass measurement process. Additionally, at each experimental point it was possible to measure the ambient temperature and atmospheric pressure in order to calculate the thrust factor due to buoyancy forces (i.e .: density difference). In this way, at each experimental point it was possible to calculate the density of the real air from the ideal gas equation. During the measurement and calibration process, the systematic error, the hysteresis error, and the expanded uncertainty associated with the mass measurement were determined at each of the 42 experimental points.
Data source location	Universidad del Atlántico, Barranquilla, Colombia.
Data accessibility	With the article

## Value of the Data

• The published data are essential to develop similar investigations that seek the application of alternative methods that allow guaranteeing the metrological reliability of mass measurement processes at an industrial level.
• The data published in this article will be useful for professionals in the area of engineering and basic sciences. In addition, metrology laboratories can make use of the data to evaluate non-parametric statistical methods applied in the calibration of digital scale.
• The published data are available to apply alternative methods for the calculation of measurement uncertainty (e.g.: Bayesian statistics, Monte Carlo method), in addition to performing the calculation by the ISO GUM method considered in OIML-R76-1. In this way, it is possible to make the calibration process of a scale for mass measurement in laboratories more robust.
• Reducing measurement uncertainty and increasing metrological reliability in mass measurement processes is a challenge for metrology and a desire for society. In this way, the evaluation and comparison of standardized methods, using the data published here –which were obtained with due metrological rigor– will allow, in the short and medium term, the updating of international standards and procedures related to the calibration of digital scales.
• The data published here has been obtained in a research laboratory under controlled environmental conditions and using calibrated measuring instruments. This guarantees the metrological traceability of the results to the international System of Units. In the same way, it is a strong indicator of the solidity and robustness of the results. Unquestionably, these data become the starting point for deeper investigations in the area of mass metrology.

## 1 Data Description

The description of the experimental data is shown in this section. To facilitate understanding, this section presents the data associated with each of the four experimentally evaluated methods. Furthermore, the supplementary material of this article presents, in detail, all the experimental data and engineering calculations that allowed to obtain all the results. Thus, an Excel calculation tool was used. This complementary file allows the reproduction of the calculations carried out in different industrial processes.

Table 1 presents the experimentally obtained data. In this Table, 42 experimental points were measured during the experiments (21 for the ascending load and 21 for the descending load situation). The table also shows the standard mass values that were used. An adequate combination of the standard masses was carried out with the objective of varying the load from 0 kg to 30 kg and, in this way, evaluating the entire operating range of the scale. Between the intermediate points the load was removed. In this way, Table 1 shows the values of 0 kg, which confirms the excellent performance of the scale in the constancy of the zero point, as recommended by OIML R-76-1 [1]. The ambient temperature and the atmospheric pressure were parameters measured for each experimental point obtained. As can be seen, the environmental conditions were controlled throughout the experimental process. Thus, the maximum variation in ambient temperature was 0.3 °C. In the atmospheric pressure there was no variation.

**Table 1:** Experimental data.

Exp. Points	Standard mass	Indicated mass by scale	Ambient Temperature	Atmospheric Pressure	Exp. Points	Standard mass	Indicated mass by scale	Ambient Temperature	Atmospheric Pressure
	kg	kg	^o^C	mbar/abs		kg	kg	^o^C	mbar/abs
1	0	0.001	27.7	1021.7	1	0	0.001	27.5	1021.7
2	3	2.997	27.7	1021.7	2	3	2.998	27.5	1021.7
3	0	0.001	27.7	1021.7	3	0	0.001	27.5	1021.7
4	6	5.993	27.7	1021.7	4	6	5.994	27.5	1021.7
5	0	0.001	27.7	1021.7	5	0	0.001	27.5	1021.7
6	9	8.998	27.7	1021.7	6	9	8.998	27.5	1021.7
7	0	0.001	27.7	1021.7	7	0	0.002	27.5	1021.7
8	12	11.977	27.7	1021.7	8	12	11.979	27.5	1021.7
9	0	0.001	27.7	1021.7	9	0	0.002	27.5	1021.7
10	15	14.964	27.7	1021.7	10	15	14.964	27.5	1021.7
11	0	0.001	27.7	1021.7	11	0	0.002	27.5	1021.7
12	18	17.996	27.7	1021.7	12	18	17.995	27.5	1021.7
13	0	0.001	27.7	1021.7	13	0	0.001	27.5	1021.7
14	21	20.992	27.7	1021.7	14	21	20.994	27.5	1021.7
15	0	0.001	27.7	1021.7	15	0	0.001	27.5	1021.7
16	24	23.994	27.7	1021.7	16	24	23.994	27.5	1021.7
17	0	0.001	27.7	1021.7	17	0	0.001	27.5	1021.7
18	27	26.989	27.6	1021.7	18	27	26.988	27.5	1021.7
19	0	0.001	27.6	1021.7	19	0	0.001	27.5	1021.7
20	30	29.987	27.5	1021.7	20	30	29.987	27.4	1021.7
21	0	0.001	27.5	1021.7	21	0	0.001	27.4	1021.7

The uncertainty analysis showed: for the situation of the first calibration method, the expanded uncertainty associated with the mass measurement is equal to 0.0012 kg as presented in Table 2. On the other hand, Table 3 presents the evaluated situation where the standard mass is not removed from the scale load cell (Method #2), obtaining an expanded uncertainty value equal to 0.025 kg. In the situation where the metrological performance of the scale was evaluated only for the ascending load (Method # 3), the expanded uncertainty associated with the measurement is equal to 0.027 kg as presented in Table 4. Finally, the metrological reliability of the scale it was evaluated only for the descending load (Method #4), the results of which are presented in Table 5. In this last evaluated situation, the expanded uncertainty associated with the measurement was equal to 0.025 kg.

**Table 2:** Uncertainty Analyses: Method #1: Ascending and descending load, returned to zero

	Exp. Point	Indicated Mass	Adjusted Mass	Adjusted Uncertainty	Uncertainty of standard mass	Uncertainty associated to resolution	Standard Uncertainty	Expanded Uncertainty (U)	Error
		kg	kg	kg	kg	kg	kg	kg	kg
**ASCENDING LOAD**	**1**	0.001	0.001	0.00020	0.00000	0.00058	0.0006	**0.0012**	**0.0000**
	**2**	2.997	3.013	0.00020	0.00000186	0.00058	0.0006	**0.0012**	**-0.0156**
	**3**	5.993	6.024	0.00020	0.0000042	0.00058	0.0006	**0.0012**	**-0.0312**
	**4**	8.998	9.045	0.00020	0.0000048	0.00058	0.0006	**0.0012**	**-0.0468**
	**5**	11.977	12.039	0.00020	0.0000087	0.00058	0.0006	**0.0012**	**-0.0623**
	**6**	14.964	15.078	0.00020	0.0000095	0.00058	0.0006	**0.0012**	**-0.1140**
	**7**	17.996	18.094	0.00020	0.0000096	0.00058	0.0006	**0.0012**	**-0.0976**
	**8**	20.992	21.109	0.00020	0.0000165	0.00058	0.0006	**0.0012**	**-0.1172**
	**9**	23.994	24.125	0.00020	0.0000167	0.00058	0.0006	**0.0012**	**-0.1308**
	**10**	26.989	27.140	0.00020	0.0000171	0.00058	0.0006	**0.0012**	**-0.1514**
	**11**	29.987	30.156	0.00020	0.0000186	0.00058	0.0006	**0.0012**	**-0.1690**

**DESCENDING LOAD**	**12**	0.000	0.000	0.00020	0.0000000	0.00058	0.0006	**0.0012**	**-0.0001**
	**13**	3.000	3.016	0.00020	0.0000019	0.00058	0.0006	**0.0012**	**-0.0156**
	**14**	6.000	6.031	0.00020	0.0000042	0.00058	0.0006	**0.0012**	**-0.0312**
	**15**	9.000	9.047	0.00020	0.0000048	0.00058	0.0006	**0.0012**	**-0.0468**
	**16**	12.000	12.062	0.00020	0.0000087	0.00058	0.0006	**0.0012**	**-0.0624**
	**17**	15.000	15.078	0.00020	0.0000095	0.00058	0.0006	**0.0012**	**-0.0780**
	**18**	18.000	18.094	0.00020	0.0000096	0.00058	0.0006	**0.0012**	**-0.0936**
	**19**	21.000	21.109	0.00020	0.0000165	0.00058	0.0006	**0.0012**	**-0.1092**
	**20**	24.000	24.125	0.00020	0.0000167	0.00058	0.0006	**0.0012**	**-0.1248**
	**21**	27.000	27.140	0.00020	0.0000171	0.00058	0.0006	**0.0012**	**-0.1404**
	**22**	30.000	30.156	0.00020	0.000019	0.00058	0.0006	**0.0012**	**-0.1560**

**Table 3:** Uncertainty Analyses: Method #2: ascending and descending load, without the need to return to zero

	Exp. Point	Indicated Mass	Adjusted Mass	Adjusted Uncertainty	Uncertainty of standard mass	Uncertainty associated to resolution	Standard Uncertainty	Expanded Uncertainty (U)	Error
		kg	kg	kg	kg	kg	kg	kg	kg
**ASCENDING LOAD**	**1**	0.001	0.007	0.01018	0.00000	0.00628	0.0120	**0.025**	**-0.0057**
	**2**	2.998	3.020	0.01018	0.00000186	0.00628	0.0120	**0.025**	**-0.0222**
	**3**	5.993	6.032	0.01018	0.0000042	0.00628	0.0120	**0.025**	**-0.0387**
	**4**	8.996	9.051	0.01018	0.0000048	0.00628	0.0120	**0.025**	**-0.0552**
	**5**	11.977	12.049	0.01018	0.0000087	0.00628	0.0120	**0.025**	**-0.0716**
	**6**	14.964	15.052	0.01018	0.0000095	0.00628	0.0120	**0.025**	**-0.0880**
	**7**	17.996	18.101	0.01018	0.0000096	0.00628	0.0120	**0.025**	**-0.1047**
	**8**	20.993	21.114	0.01018	0.0000165	0.00628	0.0120	**0.025**	**-0.1212**
	**9**	23.994	24.132	0.01018	0.0000167	0.00628	0.0120	**0.025**	**-0.1377**
	**10**	26.989	27.143	0.01018	0.0000171	0.00628	0.0120	**0.025**	**-0.1541**
	**11**	29.987	30.158	0.01018	0.0000186	0.00628	0.0120	**0.025**	**-0.1706**

**DESCENDING LOAD**	**12**	0.001	0.007	0.01018	0.0000000	0.00628	0.0120	**0.025**	**-0.0057**
	**13**	2.998	3.020	0.01018	0.0000019	0.00628	0.0120	**0.025**	**-0.0222**
	**14**	5.994	6.033	0.01018	0.0000042	0.00628	0.0120	**0.025**	**-0.0387**
	**15**	8.997	9.052	0.01018	0.0000048	0.00628	0.0120	**0.025**	**-0.0552**
	**16**	11.979	12.051	0.01018	0.0000087	0.00628	0.0120	**0.025**	**-0.0716**
	**17**	14.965	15.053	0.01018	0.0000095	0.00628	0.0120	**0.025**	**-0.0880**
	**18**	17.995	18.100	0.01018	0.0000096	0.00628	0.0120	**0.025**	**-0.1047**
	**19**	20.994	21.115	0.01018	0.0000165	0.00628	0.0120	**0.025**	**-0.1212**
	**20**	23.994	24.132	0.01018	0.0000167	0.00628	0.0120	**0.025**	**-0.1377**
	**21**	26.988	27.142	0.01018	0.0000171	0.00628	0.0120	**0.025**	**-0.1541**
	**22**	29.987	30.158	0.01018	0.000019	0.00628	0.0120	**0.025**	**-0.1706**

**Table 4:** Uncertainty Analyses: Method #3: only with ascending load

	Exp. Point	Indicated Mass	Adjusted Mass	Adjusted Uncertainty	Uncertainty of standard mass	Uncertainty associated to resolution	Standard Uncertainty	Expanded Uncertainty (U)	Error
		kg	kg	kg	kg	kg	kg	kg	kg
**ASCENDING LOAD**	**1**	0.001	0.007	0.01098	0.00000	0.00668	0.0128	**0.027**	**-0.0061**
	**2**	2.998	3.021	0.01098	0.00000186	0.00668	0.0128	**0.027**	**-0.0226**
	**3**	5.993	6.032	0.01098	0.0000042	0.00668	0.0128	**0.027**	**-0.0391**
	**4**	8.996	9.052	0.01098	0.0000048	0.00668	0.0128	**0.027**	**-0.0556**
	**5**	11.977	12.049	0.01098	0.0000087	0.00668	0.0128	**0.027**	**-0.0720**
	**6**	14.964	15.052	0.01098	0.0000095	0.00668	0.0128	**0.027**	**-0.0884**
	**7**	17.996	18.101	0.01098	0.0000096	0.00668	0.0128	**0.027**	**-0.1051**
	**8**	20.993	21.115	0.01098	0.0000165	0.00668	0.0128	**0.027**	**-0.1216**
	**9**	23.994	24.132	0.01098	0.0000167	0.00668	0.0128	**0.027**	**-0.1381**
	**10**	26.989	27.144	0.01098	0.0000171	0.00668	0.0128	**0.027**	**-0.1545**
	**11**	29.987	30.158	0.01098	0.0000186	0.00668	0.0128	**0.027**	**-0.1710**

**Table 5:** Uncertainty Analyses: Method #4: only with descending load.

	Exp. Point	Indicated Mass	Adjusted Mass	Adjusted Uncertainty	Uncertainty of standard mass	Uncertainty associated to resolution	Standard Uncertainty	Expanded Uncertainty (U)	Error
		kg	kg	kg	kg	kg	kg	kg	kg
**DESCENDING LOAD**	**1**	0.001	0.007	0.01048	0.00000	0.00588	0.0120	**0.025**	**-0.0061**
	**2**	2.998	3.021	0.01048	0.00000186	0.00588	0.0120	**0.025**	**-0.0226**
	**3**	5.994	6.033	0.01048	0.0000042	0.00588	0.0120	**0.025**	**-0.0391**
	**4**	8.997	9.053	0.01048	0.0000048	0.00588	0.0120	**0.025**	**-0.0556**
	**5**	11.979	12.051	0.01048	0.0000087	0.00588	0.0120	**0.025**	**-0.0720**
	**6**	14.965	15.053	0.01048	0.0000095	0.00588	0.0120	**0.025**	**-0.0884**
	**7**	17.995	18.100	0.01048	0.0000096	0.00588	0.0120	**0.025**	**-0.1051**
	**8**	20.994	21.116	0.01048	0.0000165	0.00588	0.0120	**0.025**	**-0.1216**
	**9**	23.994	24.132	0.01048	0.0000167	0.00588	0.0120	**0.025**	**-0.1381**
	**10**	26.988	27.143	0.01048	0.0000171	0.00588	0.0120	**0.025**	**-0.1545**
	**11**	29.987	30.158	0.01048	0.0000186	0.00588	0.0120	**0.025**	**-0.1710**

**Fig. 1:**
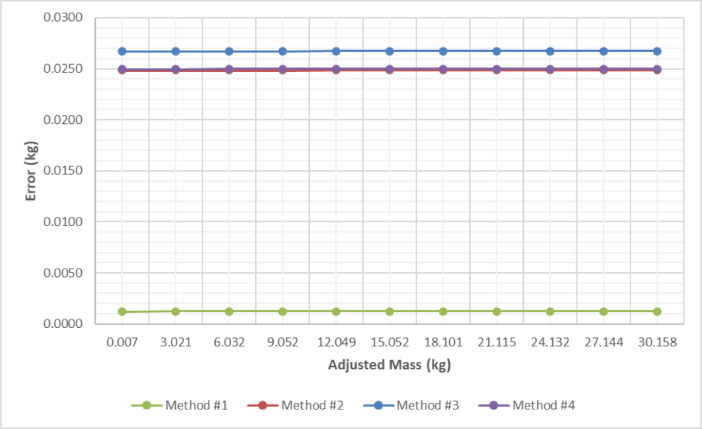
Uncertainty analyses among the different methods.

Comparing the results obtained, in terms of the expanded uncertainty associated with the mass measurement, a substantial difference is observed, mainly, when the scale is calibrated using Method #1 (*Ascending and descending load, returned to zero*). The value of the uncertainty is much lower than in the other situations. This is graphically illustrated in Fig. 1, where the horizontal axis shows the adjusted mass and the vertical axis denotes the expanded uncertainty. The differences found can be attributed to the physical nature of the problem studied, as well as the electronic operation of the scale. The experimental data documented here constitute an excellent source to continue research in the area and contribute to the advancement of knowledge in mass metrology.

## 2 Experimental Design, Materials and Methods

In the course of the experiments, a digital scale (Manufacturer: Bernalo; Model: JCS-A; Range: 0 to 30 kg; Resolution: 0.001 kg) was calibrated applying four methods proposed and described in the SIM Guide (Inter-American System of Metrology), *i.e.: (i) ascending and descending load, returned to zero; (ii) ascending and descending load, without the need to return to zero; (iii) only with ascending load and (iv) only with descending load* [2]. The specialized literature [3], [4], [5], [6] confirms that several studies have been carried out to guarantee metrological reliability in industrial measurement processes. In specific terms, in mass measurement processes, environmental conditions (atmospheric pressure and ambient temperature) strongly influence the indication of apparent mass [7,8]. Thus, the International Organization of Legal Metrology (OIML) in its recommendation R-76-1 [1] defines apparent mass as that mass value that must be corrected for environmental conditions and for gravitational effects [9,10]. Regarding the estimation of measurement uncertainty, it was obtained according to the GUM method [11].

**Fig. 2:**
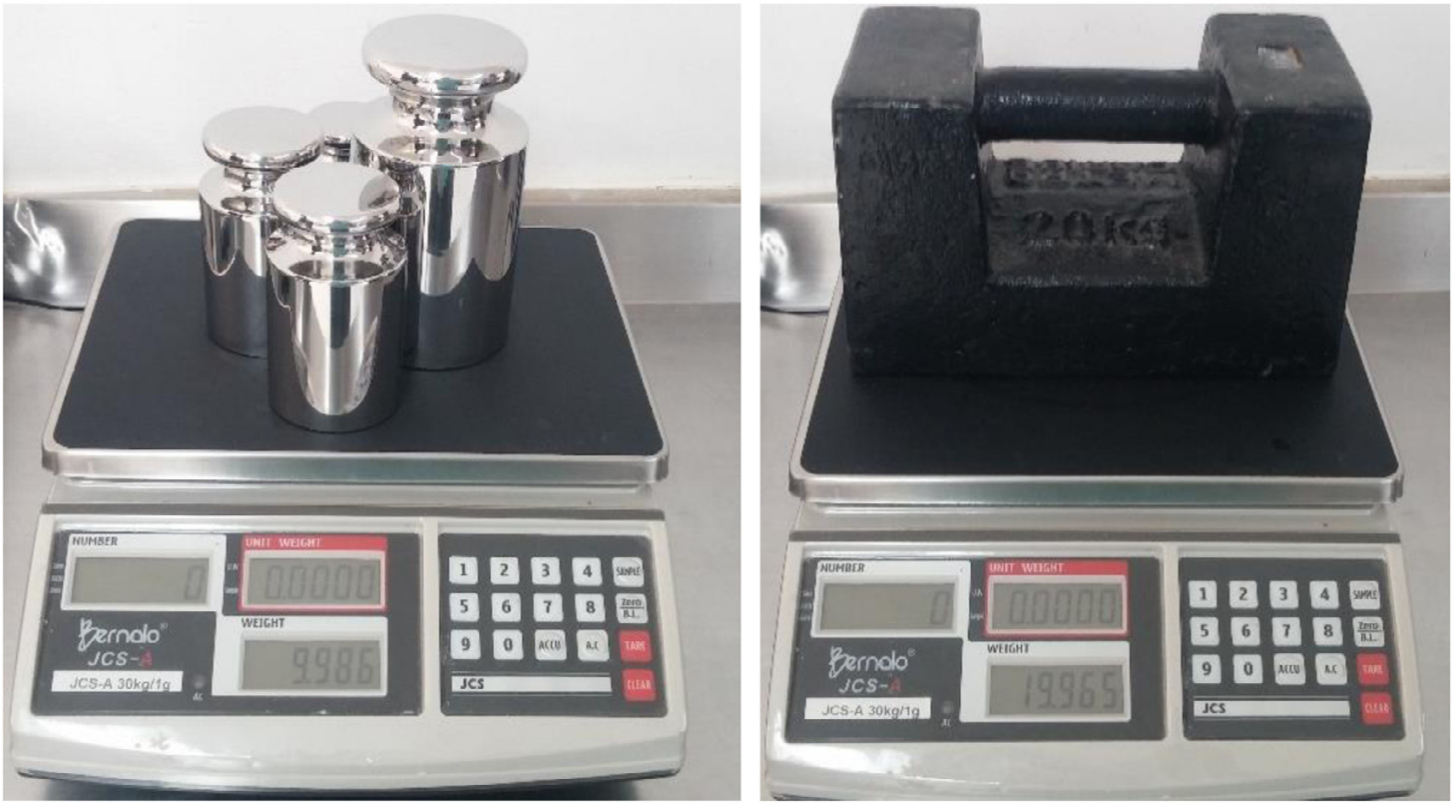
Experimental process in the calibration of a digital scale.

In relation to the development of the experiments, these were carried out in the research laboratory of the Faculty of Nutrition and Dietetics of the Universidad del Atlántico (Barranquilla-Colombia). The ambient temperature was measured with a digital thermometer (Range: 0.0 to 99.9 °C; Resolution: 0.1 °C). Atmospheric pressure was measured with a digital barometer (Range: 0.0 to 9999.9 mbar/abs; Resolution: 0.1 mbar/abs). The standard masses used in the experiments are classified as E1 and M1 according to the class established in OIML-R111 [12]. Fig. 2 illustrates the scale calibration in the experimental process.

This experimental methodology is focused, essentially, to attend the mass measurement processes in the industrial sector. As shown in this article, it is possible to clearly notice that the methods proposed by the SIM Guide can be applied with two different purposes: (i) to identify the most suitable method to calibrate a scale for the industrial sector; (ii) reduce costs associated with mass measurement. Additionally, the experimentally obtained data are used to directly evaluate the mass measurement. The literature shows that lower-range balances (up to 250 g) are used, not only for mass measurement, but also for indirect volume measurement. This is clearly shown in a paper recently published [13], where the influence of the tare function of an analytical balance for volume measurement was analysed, *i.e.*: to evaluate the influence of the tare function on a graduated cylinder of 50 ml.

## Declaration of Competing Interest

The authors declare that they have no known competing financial interests or personal relationships which have, or could be perceived to have, influenced the work reported in this article.

## Appendix Supplementary materials

Image, application 1
